# Early Onset Diffusion Abnormalities in Refractory Headache Disorders

**DOI:** 10.3389/fneur.2022.898219

**Published:** 2022-06-14

**Authors:** Jonathan D. Santoro, Peter K. Moon, Michelle Han, Emily S. McKenna, Elizabeth Tong, Sarah J. MacEachern, Nils D. Forkert, Kristen W. Yeom

**Affiliations:** ^1^Division of Neurology, Department of Pediatrics, Children's Hospital Los Angeles, Los Angeles, CA, United States; ^2^Department of Neurology, Keck School of Medicine at University of Southern California, Los Angeles, CA, United States; ^3^Stanford University School of Medicine, Stanford, CA, United States; ^4^Department of Neurology, Children's Hospital of Philadelphia, Philadelphia, PA, United States; ^5^Department of Radiology, Stanford University School of Medicine, Stanford, CA, United States; ^6^Department of Pediatrics, University of Calgary, Calgary, AB, Canada; ^7^Department of Radiology, University of Calgary, Calgary, AB, Canada

**Keywords:** migraine, MRI, diffusion imaging, chronic pain, biomarker

## Abstract

**Objective:**

This study sought to determine if individuals with medically refractory migraine headache have volume or diffusion abnormalities on neuroimaging compared to neurotypical individuals.

**Background:**

Neuroimaging biomarkers in headache medicine continue to be limited. Early prediction of medically refractory headache and migraine disorders could result in earlier administration of high efficacy therapeutics.

**Methods:**

A single-center, retrospective, case control study was performed. All patients were evaluated clinically between 2014 and 2018. Individuals with medically refractory migraine headache (defined by ICDH-3 criteria) without any other chronic medical diseases were enrolled. Patients had to have failed more than two therapeutics and aura was not exclusionary. The initial MRI study for each patient was reviewed. Multiple brain regions were analyzed for volume and apparent diffusion coefficient values. These were compared to 81 neurotypical control patients.

**Results:**

A total of 79 patients with medically refractory migraine headache were included and compared to 74 neurotypical controls without headache disorders. Time between clinical diagnosis and neuroimaging was a median of 24 months (IQR: 12.0–37.0). Comparison of individuals with medically refractory migraine headache to controls revealed statistically significant differences in median apparent diffusion coefficient (ADC) in multiple brain subregions (*p* < 0.001). *Post-hoc* pair-wise analysis comparing individuals with medically refractory migraine headache to control patients revealed significantly decreased median ADC values for the thalamus, caudate, putamen, pallidum, amygdala, brainstem, and cerebral white matter. No volumetric differences were observed between groups.

**Conclusions:**

In individuals with medically refractory MH, ADC changes are measurable in multiple brain structures at an early age, prior to the failure of multiple pharmacologic interventions and the diagnosis of medically refractory MH. This data supports the hypothesis that structural connectivity issues may predispose some patients toward more medically refractory pain disorders such as MH.

## Introduction

Migraine headache (MH) is a common and chronic condition with multi-factorial neurovascular etiologies characterized by recurrent paroxysmal attacks of throbbing headaches with or without autonomic nervous system dysfunction (1). Along with tension type-headaches (TH), migraines are one of the most frequently occurring neurologic phenomena in children and young adults (2, 3). Beyond pain, these disorders can have dramatic impacts on performance at school and work, causing marked burden of disease in patients suffering from them (4, 5).

Currently, no biomarker is available for predicting which individuals are likely to suffer from medically refractory MH. Previously, patients with MH were identified to have early cerebral diffusion abnormalities on magnetic resonance imaging (MRI) in pain sensitization regions compared to controls (6). However, that study was cross-sectional in nature and did not follow patients longitudinally to determine the outcome or severity of their headache disorders. Furthermore, there was great heterogeneity with respect to when these neuroimaging studies were acquired. Although other studies have identified late micro-structural and connectivity differences amongst individuals with vs. without MH disorders (7–10), no studies have assessed differences in severity in this population or the possibility of early prediction of refractory headache disorders based on imaging.

The aim of this study was to investigate if individuals with medically refractory MH have diffusion or volumetric abnormalities on their earliest neuroimaging study with the goal of identifying if headache disorder disease severity is associated with early neuroimaging abnormalities.

## Materials and Methods

### Standard Protocol Approvals, Registrations, and Patient Consents

All data collection, review and analysis were conducted after approval by the Stanford University institutional review board (No. 36206).

### Data Availability

All data is available in an anonymized format to qualified investigators following release approval by the institutional review board.

### Study Design

Retrospective, cross-sectional. Patients who had been diagnosed with medically refractory headache disorders were retrospectively assessed for imaging abnormalities on their first lifetime neuroimaging (MRI) study. Imaging data was extracted from the first lifetime neuroimaging study but clinical data was only extracted from the last clinical encounter to ensure capture of most recent headache-related diagnosis.

### Inclusion Criteria

Inclusion for the study cohort were: a diagnosis of MH as defined by the International Classification of Headache Disorders Version 3.0 (ICHD-3). (1) patients were sub-grouped into episodic MH (EMH) and chronic (CMH) and MH with or without neurologic aura per ICHD-3 criteria. All types of aura were included for the purposes of this study. All patients required neuroimaging within 18 months of the MH diagnosis, which was acquired on a 3T MRI scanner at the institutions mentioned above for consistency purposes. Prior neuroimaging findings must have been clinically interpreted as normal, which excluded any patients with incidental findings or abnormalities (e.g., T2 signal prolongation of unknown significance—also known as unidentified bright objects), developmental venous anomaly, Chiari I abnormality, etc.).

Control subjects obtained brain MRI at 3T as part of standard of care for evaluation and interpreted by board-certified neuroradiologists to have normal exam. A comprehensive manual chart review was performed to ensure no prior history MH or underlying neurologic, cognitive, or neuropsychiatric disorders, as well as cancer history, or other clinical diseases requiring chronic medical therapies, chemotherapy, or radiation. Clinical reasons for imaging included syncope, nausea, scalp nevus, cholesteatoma, sinus disease, orbital strabismus, and family history of aneurysm, vascular malformation, or cancers. All included cases were reviewed by two authors. In cases where inclusion was discrepant, the senior author served as an arbiter for inclusion/exclusion.

### Exclusion Criteria

Strict exclusion criteria were applied and comprised the following: inadequate data or image-registration quality, any concern for co-morbid secondary headache (e.g., use of non-headache related pharmacotherapy with side effect of headache), current or prior history of developmental delay or intellectual disability, history of or active medication-overuse headache, tension-type headache, underlying cardiac disease, underlying pulmonary disease, epilepsy, prior or current hemorrhage, vascular lesions (aneurysm, AVM, fistula, or steno-occlusive disease), or prior strokes, given their potential impact on regional diffusion properties in the brain. Patients were permitted to have failed no greater than one preventative headache therapies prior to neuroimaging. Additionally, any patient with a previously diagnosed genetic, metabolic, or chronic medical disease was excluded from this study. Patients with any focal neurologic findings, even if incidental, were excluded. Patients with incomplete or inconsistent data were also excluded. Patients who had an active headache or migraine at the time of neuroimaging (as documented on the day of encounter on a screening form administered by the radiology technician) were excluded. All included cases were reviewed by two authors. In cases where inclusion was discrepant, the senior author served as an arbiter for inclusion/exclusion.

### Clinical Data Collection

All data were collected retrospectively by manual chart review and medication ordering summaries. Clinically related headache data included family history of headache in a first degree relative, age of onset, the number and types of therapies failed, if opioids were utilized at any time point, ICDH-3 diagnosis type (including status of aura), and medications used at the time of imaging. Medically refractory was determined as having failed at least two preventative headache therapies of three different classes, consistent with the American Headache Society criteria (11). In patients with serial neuroimaging, only the first neuroimaging study was examined for the purposes of this study. Time to last headache was not collected as this was not feasible for the retrospective review, but patients' MR imaging intake records were reviewed to exclude patients with active headache or migraine.

### MR Imaging Acquisition

All subjects underwent brain MRI at 3T (Discovery 750W; GE Healthcare, Milwaukee, Wisconsin) with an 8-channel head coil on a single MR imaging scanner. Echo-planar whole-brain diffusion-weighted MRI (DWI) was acquired in all cases with repetition time (TR) = 1,500 ms, echo time (TE) = 37 ms, flip angle = 90°, FOV = 24 cm^2^, acceleration factor = 2, in-plane resolution = 0.94 mm^2^, acquisition matrix = 128 × 128 interpolated to a 256 × 256 matrix, 44 sections with 4-mm slice thickness, no skip, two diffusion-weightings of b = 0 s/mm^2^ and b = 1,000 s/mm^2^, with diffusion gradients acquired in 3 directions averaged for the latter. Apparent diffusion coefficient (ADC), derived from DWI, has demonstrated high reproducibility and was performed as part of routine institutional neuroimaging (12). Documentation of imaging encounters were reviewed to ensure patients had no active headache or migraine at the time of scan.

### Image Processing

A custom image-processing pipeline was used in this work to extract quantitative values of regional brain volume and ADC values, previously described in more detail by Forkert et al. (13) Briefly described, after motion correction of the DWI dataset acquired with and without diffusion-weighting using rigid registration, the quantitative apparent diffusion coefficient parameter map was calculated by applying the Stejskal-Tanner equation. For regional diffusion and volumetric analysis, the Montreal Neurological Institute-152 brain atlas was non-linearly registered to the DWI dataset and the resulting transformation was used to warp the Harvard-Oxford subcortical atlas brain regions to the subject-specific brain anatomy (14). The Harvard-Oxford brain regions warped to the DWI datasets were used directly for volume assessment and calculation of median ADC values to ensure that the volume and ADC measurements are based on exactly the same brain regions. Gray matter images were unmodulated. Brain regions included in this brain atlas are the cerebral cortex, cerebral white matter, thalamus, caudate, putamen, globus pallidus, amygdala, hippocampus, brain stem, and nucleus accumbens. Two experienced observers (NDF and KWY) checked all registration results to ensure suitable data and registration quality. The aligned brain atlas regions were then used to measure the corresponding regional brain volumes and median ADC values combined for corresponding brain structures in the left and right hemispheres, whereas the lateral ventricles were only used for volumetric assessment.

### Statistical Analysis

Multivariate analysis of covariance (MANCOVA) was used for group comparison of the control group and individuals with MH using the volumetric and median ADC values as dependent variables, age as a covariate, duration of symptoms (episodic/chronic) and the class (MH) as the fixed factor. To assess whether diffusion metrics are predictive of migraine status, simple and multiple logistic regression models were constructed. SPSS (Version 24.0, IBM, Armonk, NY) was used for MANCOVA statistical analyses. Graphpad Prism (Version 9.1.1) was used for regression analysis. A *P*-value < 0.05 (Bonferroni-corrected) was considered significant. To minimize risk of Type 1 error that can occur in the setting of multiple comparisons, we employed the most conservative Bonferroni correction for all of our analyses.

For analyses involving volumetric data, we corrected for 11 tests given that we ran tests for 11 brain regions. For analyses involving diffusion, we corrected for 10 tests given that we ran tests for 10 brain regions.

## Results

In total, 112 patients met inclusion criteria. Thirty-three of these patients met at least one exclusion criteria, leading to 79 patients for analysis (70%). Of those excluded, the most frequent reasons were incomplete data (*n* = 15), diagnosis of an alternative exclusionary type of headache (*n* = 8), incidental imaging finding (*n* = 4), and corrupted imaging sequences (*n* = 4). Cohen's kappa for inter-rate agreement for application of inclusion/exclusion was 0.99 (one disagreement). A total of 84 patients were identified for the control arm of this study with 74 (88%) meeting no exclusionary criteria. Demographic data is presented in Table 1. The median age of the MH cohort was 22.3 (IQR: 17.5–26.5) compared to the control cohort with a median age of 20.5 years (IQR: 16.0–26.0). Seventy-seven percent (*n* = 61) of patients in the MH group were female compared to 67% (*n* = 50) in the control group. Patients with MH were classified as either episodic or chronic per ICDH-3 criteria (1). Thirty-seven patients had episodic MH and 42 patients had chronic MH. Ten patients with episodic MH had associated aura compared with 14 chronic MH patients. The median age of onset for patients was 13 years (IQR: 10.0–13.0) with the median time between diagnosis and first MRI being 24 months (IQR: 12.0–37.0).

**Table 1:** Clinical and Demographic Data.

**Characteristics**	**Control** **(*n* = 74)**	**Migraine** **(*n* = 79)**
Median age (IQR: 25th– 75th)^a^	20.5 (16.0–26.0)	22.3 (17.5–26.5)
Median age at onset (IQR: 25th−75th)		13.0 (10.5–15.0)
Sex (*n*, %)^b^	24 (32.4)	18 (22.8)
M		
F	50 (67.6)	61 (77.2)
First degree relative with migraine		34 (43.0)
Migraine Type—no. (%)		27 (34.2)
Episodic without Aura		
Episodic with Aura		10 (12.7)
Chronic without Aura		28 (35.4)
Chronic with Aura		14 (17.7)
Median number of medications tried (IQR: 25th−75th)		2.0 (0.5–3.5)
Episodic without Aura		
Episodic with Aura		3.5 (0.5–7.3)
Chronic without Aura		12.0 (7.5–14.0)
Chronic with Aura		15.5 (8.3–19.5)

a *T-value = 1.175, P-value = 0.242*.

b *Fisher's exact test P-value = 0.207*.

**Figure 1:**
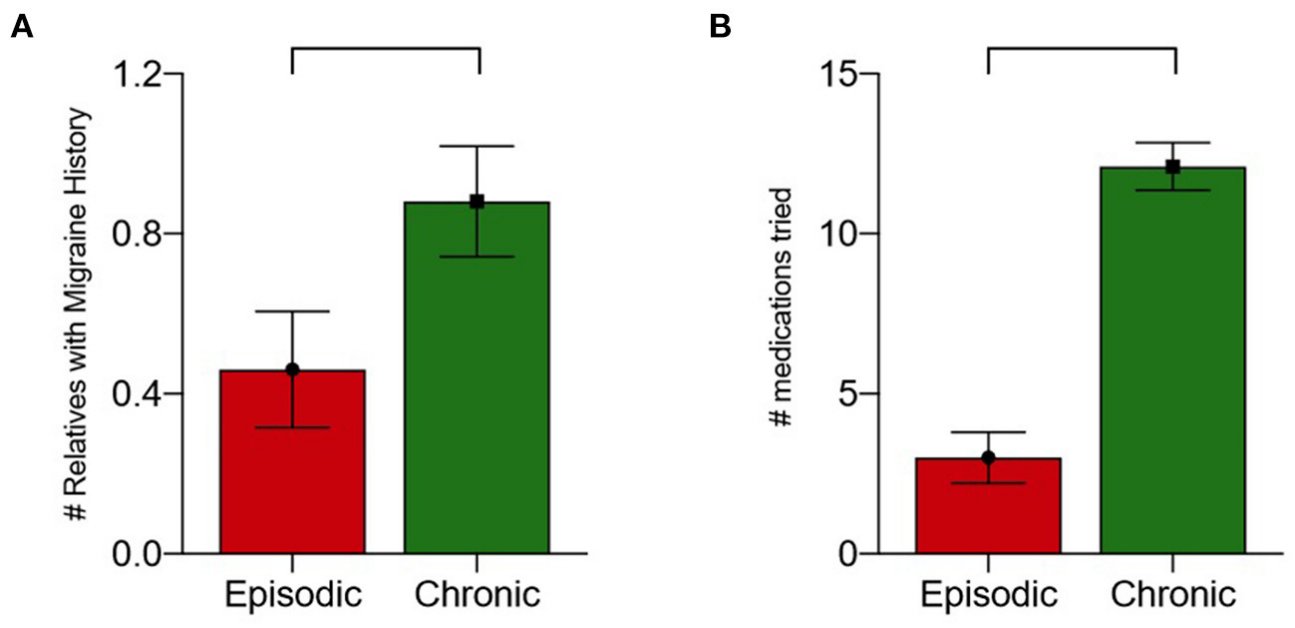
**(A)** Number of first-degree relatives with migraine history by migraine type with correction for age and sex (*p* = 0.035). **(B)** Number of medications tried by migraine type (*p* < 0.001).

There was a statistically significant difference between the average number of first-degree relatives with MH who had EMH (0.43) and CMH (0.76) after controlling for the covariate effects of age and sex (*p* = 0.034, 95^th^% CI: 10.6–13.5, Figure 1A). Neither age nor sex was significantly related to the number of relatives with migraine history.

The median number of medication failures for all patients was 6 (IQR: 3.0–12.0). Patients with EMH without aura tried a median of 2.0 medications, while those with aura tried a median of 3.5 medications. Patients with CMH without aura tried a median of 12.0 medications while those with aura tried a median of 15.5 medications (Figure 1B). There was a statistically significant difference between the number of medications tried by individuals with EMH compared to CMH after controlling for the covariate effects of age and sex (*p* < 0.001, 95% CI: 0.17–0.74). Neither age nor sex were found to be significant covariates regarding to the number of medications tried.

**Figure 2:**
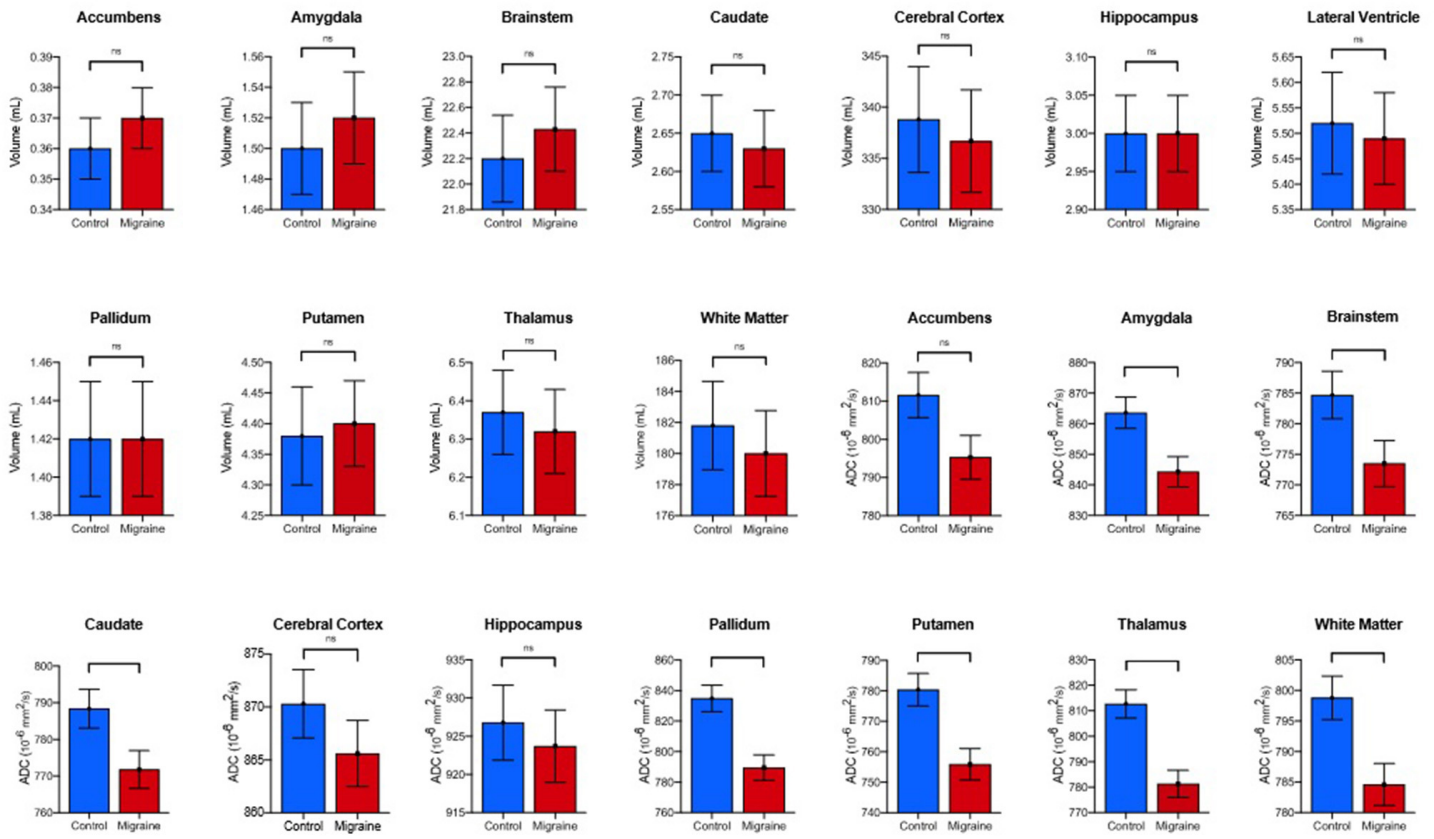
Comparison of estimated marginal means and standard error means of volume and diffusion in individuals with MTH vs. controls. *P*-values and significance generated from *post-hoc* pair-wise analysis as denoted as follows: ns, *p < 0.05, **p < 0.01, ***p < 0.001 (Bonferroni-corrected).

**Figure 3:**
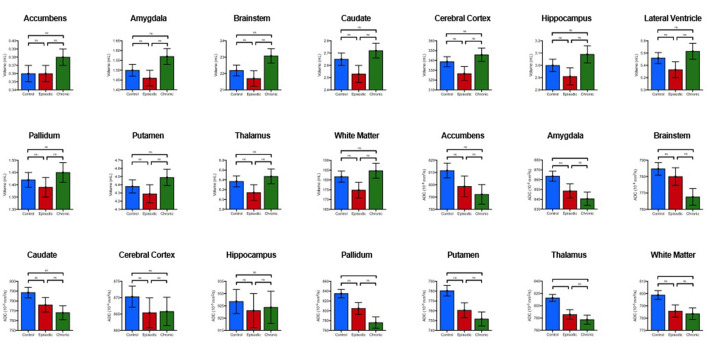
Comparison of estimated marginal means and standard en-or means of volume and diffusion amongst chronic MTH, episodic MTH and controls. *P*-values and significance generated from *post-hoc* pair-wise analysis as denoted as follows: ns, *p < 0.05, **p < 0.01, ***p < 0.001 (Bonferroni-corrected).

Comparison of individuals with MH to controls revealed statistically significant differences in median ADC in multiple brain subregions (*p* < 0.001, Figures 2, 3). *Post-hoc* pair-wise analysis comparing the migraine to control patients revealed significantly decreased median ADC values for the thalamus, caudate, putamen, pallidum, amygdala, brainstem, and cerebral white matter (Table 2). The nucleus accumbens, cerebral cortex and hippocampus did not display statistically significant differences in ADC although similar trends in lower ADC were present in individuals with migraine. Simple and multiple logistic regression models were constructed to assess predictive ability of diffusion in the seven significant brain regions, though area under the ROC curve (AUC) values associated with these models were suggestive of poor discrimination (range: 0.55–0.70) (Supplementary Figure S1). No volumetric differences were observed between groups.

**Table 2:** Volumetric and apparent diffusion coefficient values by brain region.

	**Control (*****N*** **= 74)**				**Migraine (*****N*** **= 79)**				**Univariate test**	
	**Mean^a^**	**SE**	**Lower 95% CI**	**Upper 95% CI**	**Mean^a^**	**SE**	**Lower 95% CI**	**Upper 95% CI**	**Mean Diff**.	***p*** **-value^b^**
**Volume (mL)**										
Cerebral white matter	181.8	2.85	176.1	187.4	180.0	2.76	174.5	185.4	−1.78	0.656
Cerebral cortex	338.8	5.17	328.6	349.0	336.7	5.00	326.9	346.6	−2.05	0.777
Lateral ventricle	5.52	0.10	5.33	5.70	5.49	0.09	5.31	5.67	−0.03	0.833
Thalamus	6.37	0.11	6.15	6.59	6.32	0.11	6.10	6.53	−0.06	0.728
Caudate	2.65	0.05	2.55	2.75	2.63	0.05	2.53	2.72	−0.02	0.731
Putamen	4.38	0.08	4.23	4.53	4.40	0.07	4.25	4.54	0.02	0.873
Pallidum	1.42	0.03	1.36	1.47	1.42	0.03	1.37	1.47	0.00	0.943
Hippocampus	3.00	0.05	2.90	3.10	3.00	0.05	2.91	3.10	0.00	0.955
Amygdala	1.50	0.03	1.44	1.55	1.52	0.03	1.46	1.57	0.02	0.641
Accumbens	0.36	0.01	0.35	0.38	0.37	0.01	0.35	0.39	0.00	0.760
Brainstem	22.20	0.34	21.53	22.86	22.43	0.33	21.79	23.08	0.23	0.622
**Median ADC (10**^**−6**^ **mm**^**2**^**/s)**
Cerebral white matter	798.8	3.57	791.7	805.8	784.6	3.45	777.8	791.5	−14.15	**0.005** ^c^
Cerebral cortex	870.3	3.23	863.9	876.7	865.6	3.13	859.5	871.8	−4.64	0.307
Thalamus	812.7	5.51	801.8	823.6	781.3	5.33	770.8	791.8	−31.41	**<0.001** ^c^
Caudate	788.4	5.33	777.9	799.0	771.8	5.15	761.6	782.0	−16.63	**0.027** ^c^
Putamen	780.4	5.34	769.9	791.0	755.9	5.17	745.7	766.1	−24.53	**0.001** ^c^
Pallidum	834.9	8.68	817.7	852.1	789.5	8.40	772.9	806.1	−45.40	**<0.001** ^c^
Hippocampus	926.8	4.90	917.1	936.5	923.7	4.74	914.4	933.1	−3.03	0.659
Amygdala	863.6	5.09	853.5	873.7	844.3	4.93	834.6	854.1	−19.26	**0.008** ^c^
Accumbens	811.6	5.94	800.0	823.3	795.3	5.75	783.9	806.6	−16.33	0.051
Brainstem	784.7	3.88	777.0	792.4	773.5	3.76	766.1	780.9	−11.22	**0.041** ^c^

a *Covariates appearing in the model are evaluated at the following values: age (years) at time of MRI = 21.71, sex (1 as female) = 0.73*.

b *Based on the linearly independent pairwise comparisons among the estimated marginal means*.

c *P < 0.05 for statistical significance, Bonferroni-corrected. Bolded items correspond to a p values < 0.05*.

Secondary analyses revealed differences between MH sub-groups. Compared to controls, patients with CMH had lower median ADC values in the thalamus, putamen, pallidum, amygdala, brainstem, and cerebral white matter. Compared to controls, individuals with EMH showed higher median ADC in the thalamus (Table 3). There were no statistically significant ADC differences between individuals with CMH and EMH. No volumetric differences were observed between MH sub-groups and controls. Individuals with or without aura did not show any statistically significant diffusion differences, either when compared to each other or sub-type (e.g., EMH with aura vs. EMH without aura).

**Table 3:** Volumetric and apparent diffusion coefficient values by migraine type and brain region.

	**Control** **(*****N*** **= 74)**		**Episodic migraine** **(*****N*** **= 37)**		**Chronic migraine** **(*****N*** **= 42)**		**Univariate test** ^b^		
	**Mean** ^a^	**SE**	**Mean** ^a^	**SE**	**Mean** ^a^	**SE**	***p*** **-value** ^c^	***p*** **-value** ^d^	***p*** **-value** ^e^
**Volume (mL)**									
Cerebral white matter	181.7	2.83	174.8	3.98	184.7	3.77	0.469	1.000	0.218
Cerebral cortex	338.7	5.12	326.6	7.21	345.8	6.81	0.520	1.000	0.164
Lateral ventricle	5.52	0.09	5.33	0.13	5.63	0.13	0.746	1.000	0.286
Thalamus	6.37	0.11	6.14	0.16	6.47	0.15	0.712	1.000	0.379
Caudate	2.65	0.05	2.53	0.07	2.72	0.06	0.441	1.000	0.141
Putamen	4.38	0.08	4.29	0.11	4.49	0.10	1.000	1.000	0.488
Pallidum	1.42	0.03	1.39	0.04	1.45	0.04	1.000	1.000	0.864
Hippocampus	3.00	0.05	2.91	0.07	3.09	0.07	0.864	0.834	0.180
Amygdala	1.50	0.03	1.46	0.04	1.57	0.04	1.000	0.425	0.152
Accumbens	0.36	0.01	0.36	0.01	0.38	0.01	1.000	1.000	0.698
Brainstem	22.19	0.33	21.70	0.47	23.08	0.44	1.000	0.338	0.101
**Median ADC (10**^**−6**^ **mm**^**2**^**/s)**									
Cerebral white matter	798.8	3.58	785.8	5.03	783.6	4.76	0.113	**0.036** ^f^	1.000
Cerebral cortex	870.3	3.23	865.4	4.56	865.8	4.31	1.000	1.000	1.000
Thalamus	812.7	5.52	785.8	7.76	777.3	7.34	**0.016** ^f^	**<0.001** ^f^	1.000
Caudate	788.5	5.33	776.0	7.50	768.0	7.09	0.537	0.071	1.000
Putamen	780.4	5.35	760.6	7.53	751.7	7.11	0.099	**0.005** ^f^	1.000
Pallidum	835.0	8.62	804.7	12.13	776.0	11.47	0.131	**<0.001** ^f^	0.262
Hippocampus	926.8	4.91	923.1	6.92	924.4	6.54	1.000	1.000	1.000
Amygdala	863.6	5.10	848.6	7.18	840.6	6.78	0.269	**0.023** ^f^	1.000
Accumbens	811.6	5.96	798.7	8.38	792.2	7.92	0.637	0.159	1.000
Brainstem	784.7	3.86	780.0	5.43	767.6	5.14	1.000	**0.027** ^f^	0.296

a *Covariates appearing in the model are evaluated at the following values: age (years) at time of MRI = 21.71, sex (1 as female) = 0.73*.

b *Based on the linearly independent pairwise comparisons among the estimated marginal means*.

c *Episodic—Control*.

d *Chronic—Control*.

e *Chronic—Episodic*.

f *P < 0.05 for statistical significance, Bonferroni-corrected. Bolded items correspond to a p values < 0.05*.

## Discussion

**Figure 4:**
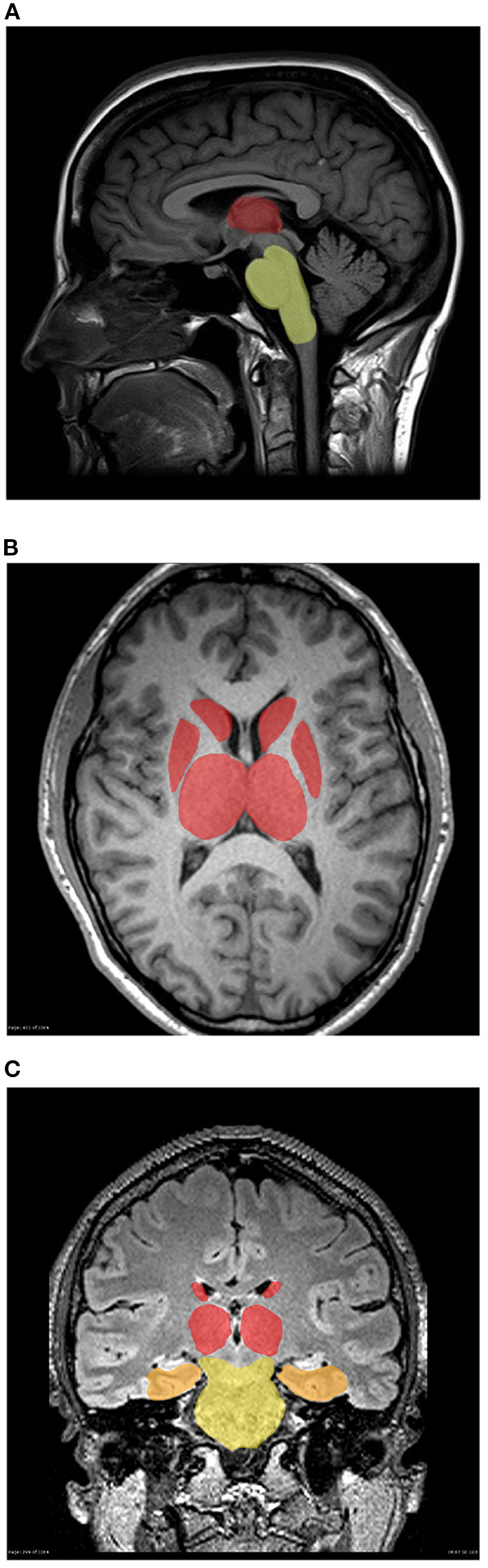
**(A–C)** Three-dimensional mapping of deep brain structure demonstrating gradient of smallest to greatest diffusion abnormalities in individuals with migraine vs. controls **(A)** Sagital, **(B)** Axial, and **(C)** Coronal. Brainstem (yellow), amygdala (orange), thalamus, putamen and pallidum (red).

This study builds on prior work identifying early ADC diffusion changes in several areas of the limbic and pain systems of the central nervous system in patients with migraine (Figure 4) (6). The main contribution of this study is the finding that in individuals with medically refractory MH, these changes are measurable in additional brain structures at an early age, *prior to* the failure of multiple pharmacologic interventions and the diagnosis of medically refractory MH. This data supports the hypothesis that structural connectivity issues may predispose some patients toward more medically refractory pain disorders such as MH.

Diffusion abnormalities of the limbic and pain sensitization structures (thalamus, caudate, putamen, pallidum, amygdala, brainstem, and cerebral white matter) were observed in individuals with MH. These structures have been previously implicated in resting-state functional MRI studies in individuals with CMH, each with a unique role in pain processing (15). However, functional neuroimaging is not a clinical standard of care, highlighting the importance of easy-to-use methods of assessment reported in this study which can be added on to routine neuroimaging sequences with minimal cost. Regression analysis did not reveal a predictive model for type of headache or migraine diagnosis later in life although sub-group analysis limited the power to assess these findings. Ultimately, greater patient volume in each group will be needed to power a clinical predictive measure that could inform clinical decision making and is an area under evaluation by the research team.

The neuroanatomic regions identified as abnormal in this study each have independent yet interlinked roles in pain processing. The amygdala has been implicated in pro-nociceptive functionality in addition to perpetuating the cortex-driven pain association (16–19). It has been reported that prolonged potentiation of the nociceptive information can be caused by aberrant activation of the amygdala (20), potentially providing insight into those at risk for more medically refractory pain disorders. Thalamic activation in pain is widely recognized. Multiple studies have identified abnormal connectivity of the thalamus to limbic and cortical structures in individuals with MH (19, 21–23). It has also been hypothesized that the neurocognitive impact of migraines (extreme fatigue, poor concentration, and sensitivity to external stimuli) may also be implicated through dysfunctional thalamic circuitry (19, 23, 24). Due to their high connectivity, thalamic circuits are tightly interwoven with the structures of the basal ganglia and brainstem, which have all also been implicated in chronic pain disorders (19, 25–29). Although the significance of diffusion abnormalities in the cerebral white matter is more difficult to ascertain, there is emerging data that insular white matter may be implicated in increased nociceptive perception as well (19, 30, 31). Although this study was underpowered to evaluate differences between patients with and without aura, individuals with MH have been hypothesized to have white matter insult caused by microvascular ischemic changes, which occur during migraines (32). Although the complexities of pain and nociception in the CNS are extraordinarily complex, this study identifies abnormalities in nearly all of these structures in individuals with the most severe MH, highlighting the possible utility of diffusion neuroimaging to identify patients at risk for medically refractory courses at an early stage. Although further study would be needed, it could be hypothesized that this group of individuals may benefit from more early and aggressive therapeutic interventions.

Individuals with CMH had lower median ADC values in the thalamus, putamen, pallidum, amygdala, brainstem, and cerebral white matter compared to EMH and controls. These findings are clinically relevant for two reasons. First, it indicates that the neuroimaging abnormalities found in patients with MH may be mostly driven by individuals with CMH. Second, it highlights that pain sensitization centers such as the thalamus, amygdala, and brainstem show micro-structural changes as measured by DWI in individuals with severe and chronic disease even at an early age. It is possible that these areas may also show atrophy (macro-structural changes) over time (33) as well although given the infrequency of clinically indicated repeat neuroimaging in patients with well-established CMH, this analysis was not feasible for this study.

This study is not without limitations. First, this is a retrospective study analyzing patients for imaging abnormalities after their diagnosis has been made, introducing the possibility of observer bias. This was mitigated by having the clinical data extraction and analysis be performed independently by the authors. This study was performed using single-center data, which may limit its generalizability. As a retrospective chart-based review, there is the risk of diagnostic inaccuracy with regards to type of MH although this was mitigated by ensuring all patients met ICHD-3 criteria. In addition, patients had been seen by multiple providers who had different mechanisms of reporting clinical features of their patients and as such, data on clinical phenotypes was far too incomplete for analysis. As previous studies have indicated that diffusion findings may be transient in adults, whether the patient was having a headache at the time of scanning or near the time of scanning may affect the quantitative imaging findings (34–36). The authors did note that total number of failed pharmacotherapeutics was higher in the CMH group. This is logical given the greater disease burden although the authors cannot rule out an impact on total pharmacotherapy exposure and longstanding diffusion changes. It is impossible to rule out the effect of active or previously attempted pharmacotherapy on neuroimaging findings in this study. The authors attempted to sub-analyze individuals with similar active and historical treatments but given the heterogeneity in headache management, total patients in each group was well below any ability to statistically analyze. Prospective studies will be needed to address this variable. Although an ideal comparator group for patients with medically refractory headaches would have been patients with medication responsive headaches, there was insignificant numbers of individuals with early neuroimaging in this latter group. In addition, severity bias in these individuals would have interfered with interpretation of data. Future, prospective studies, by the authorship group will focus having a dedicated “non-refractory” group to serve as an additional comparator arm to neurotypical controls. Further, this study did not evaluate neuroimaging findings in other individuals with chronic, non-headache, pain disorders. While the findings in this study are presumed to be specific to headache and migraine, it is possible that other chronic pain disorders (e.g., fibromyalgia) could produce similar findings and is a logical next step for this study group to investigate. For this analysis, we combined data from both the left and right hemispheres to reduce the number of hypotheses tested, which omitted trends in laterality. In addition, we recognize that segmentation of small cerebral structures can be imperfect, but an automated approach (with visual quality control) was used to ensure reproducibility, given that manual segmentation is prone to observer bias. Another limitation of this study is that we did not compare this data of individuals with MH to patients with TH. Given the lower acuity, infrequency of utilization of preventative pharmacotherapy, infrequency of neuroimaging, and higher likelihood of mixed or secondary headaches, such an analysis of this population would be inferior for the purposes of identifying imaging abnormalities in the most medically refractory headache disorders. Finally, we excluded four patients for having incidental neuroimaging findings (all punctate T2 signal prolongations of unclear significance), which may have skewed our severity toward less impacted individuals.

## Conclusions

This study identifies early cerebral diffusion changes in individuals with medically refractory MH compared to healthy controls years before therapeutic failures. The hypothesized underlying pathophysiologic mechanisms of nociception and pain sensitization in MH are probable explanations for the observed neuroimaging abnormalities. Further study is needed to investigate the predictive value of these identified diffusion abnormalities.

## Data Availability Statement

The raw data supporting the conclusions of this article will be made available by the authors, without undue reservation.

## Ethics Statement

The studies involving human participants were reviewed and approved by Stanford University: 0456218. Written informed consent from the participants' legal guardian/next of kin was not required to participate in this study in accordance with the national legislation and the institutional requirements.

## Author Contributions

JS and PM were responsible for drafting and revision of the manuscript for content, including medical writing for content. He had a major role in the acquisition of data. He was responsible for study concept and design and analysis and interpretation of data. MH was responsible for acquisition of the data, analysis, and interpretation of the data. EM played a major role in the acquisition of the data and assisted with the interpretation of data. She also revised and edited the manuscript for intellectual content. ET was responsible for drafting and revising the manuscript for intellectual content and supervised data collection. She also assisted with analysis and interpretation of the data. SM was responsible for study concept and design. She assisted with analysis and interpretation of the data and assisted with revision and editing of the manuscript for intellectual content. NF was responsible for drafting and revision of the manuscript for content, including medical writing for content. He had a major role in the acquisition of data and supervised data collection. He was responsible for study concept and design and analysis and interpretation of data. KY was responsible for drafting and revision of the manuscript for content, including medical writing for content. She had a major role in the acquisition of data and supervised data collection. She was responsible for study concept and design and analysis and interpretation of data. All authors contributed to the article and approved the submitted version.

## Conflict of Interest

The authors declare that the research was conducted in the absence of any commercial or financial relationships that could be construed as a potential conflict of interest.

## Publisher's Note

All claims expressed in this article are solely those of the authors and do not necessarily represent those of their affiliated organizations, or those of the publisher, the editors and the reviewers. Any product that may be evaluated in this article, or claim that may be made by its manufacturer, is not guaranteed or endorsed by the publisher.

## Abbreviations

ADC: Apparent diffusion coefficient
CMH: Chronic migraine headache
DWI: Diffusion weighted imaging
EMH: Episodic migraine headache
ICHD-3: International Classification of Headache Disorders Version 3.0
MRI: Magnetic resonance imaging
MANCOVA: Multivariate analysis of covariance
MH: Migraine headache
TH: Tension headache.
