# Supplementary material for: Early Onset Diffusion Abnormalities in Refractory Headache Disorders
Source: Front Neurol. 2022 Jun 14;13:898219. doi: 10.3389/fneur.2022.898219 (PMC9237368; doi:10.3389/fneur.2022.898219)
Supplement: Supplementary file 1 [file Data_Sheet_1.PDF]

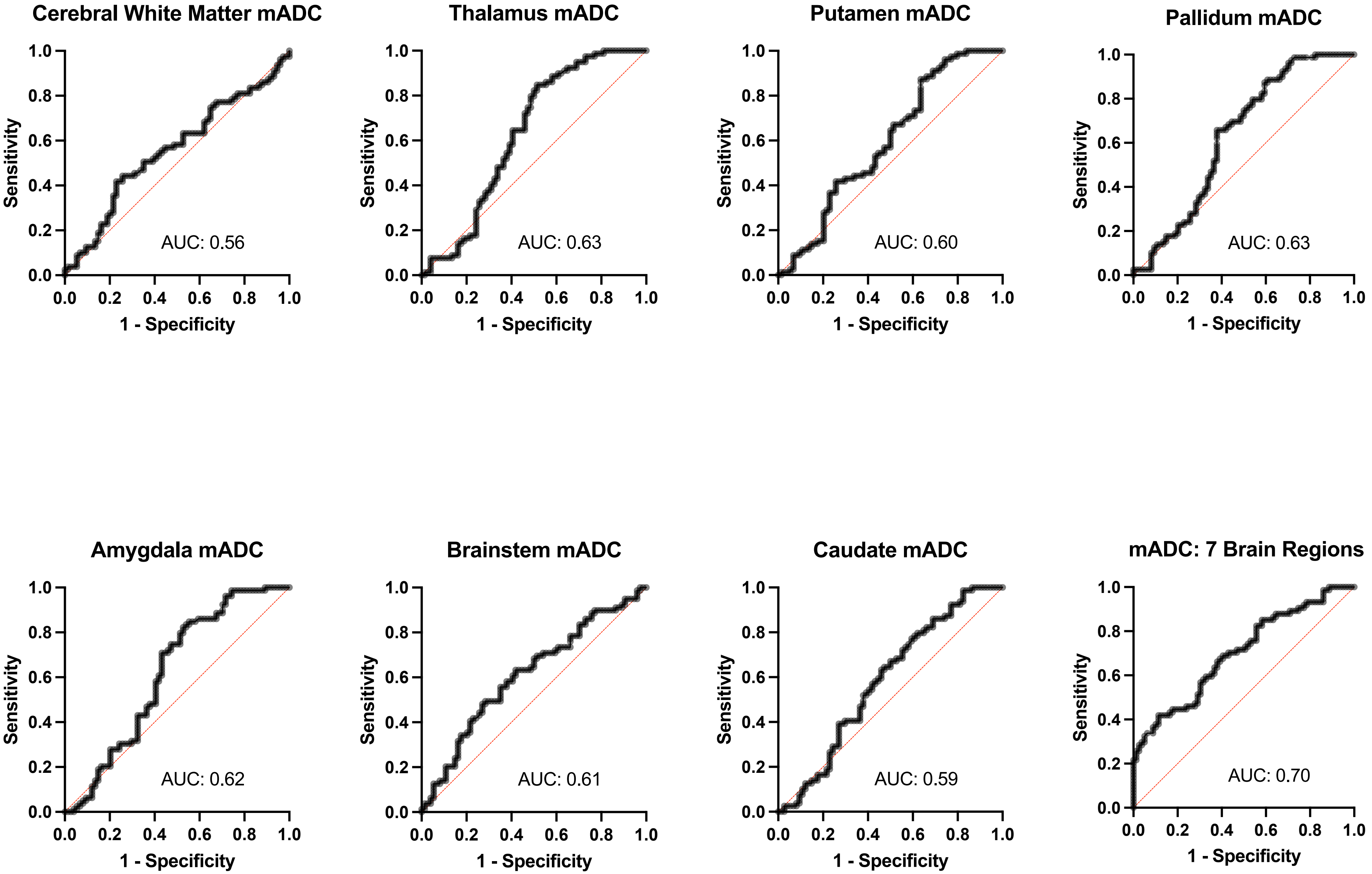
Cerebral White Matter mADC
AUC: 0.56
Thalamus mADC
AUC: 0.63
Putamen mADC
AUC: 0.60
Pallidum mADC
AUC: 0.63
Amygdala mADC
AUC: 0.62
Brainstem mADC
AUC: 0.61
Caudate mADC
AUC: 0.59
mADC: 7 Brain Regions
AUC: 0.70
Sensitivity
1 - Specificity
0.0
0.2
0.4
0.6
0.8
1.0
